# Automatic Respiratory and Bulk Patient Motion Corrected 3D Fetal MRI

**DOI:** 10.1002/mrm.70166

**Published:** 2025-11-04

**Authors:** Robin Ferincz, Leonor Alamo, Aurelio Secinaro, Estelle Tenisch, Aurélien Bustin, Davide Piccini, Milan Prša, Jérôme Yerly, Christopher W. Roy

**Affiliations:** ^1^ Department of Radiology Lausanne University Hospital (CHUV) and University of Lausanne (UNIL) Lausanne Switzerland; ^2^ Department of Radiology and Interventional Radiology Lausanne University Hospital and University of Lausanne Lausanne Switzerland; ^3^ Advanced Cardiothoracic Imaging Unit, Department of Imaging Bambino Gesù Children's Hospital IRCCS Rome Italy; ^4^ IHU LIRYC, Electrophysiology and Heart Modeling Institute Université de Bordeaux, INSERM, Centre de Recherche Cardio‐Thoracique de Bordeaux Pessac France; ^5^ Center for Biomedical Imaging (CIBM) Lausanne Switzerland; ^6^ Scientific Collaborations and Strategic Partnerships, Siemens Healthcare Srl Milano Italy; ^7^ Woman‐Mother‐Child Department Lausanne University Hospital and University of Lausanne Lausanne Switzerland

**Keywords:** 3D, fetal, free‐running, motion correction, radial, simulation

## Abstract

**Purpose:**

To develop a framework (ACROBATIC) for correcting motion in 3D radial fetal MRI.

**Methods:**

Data were simulated (*N* = 200) and acquired in utero (*N* = 11, gestational age: 32 ± 2 weeks). Motion due to maternal respiration was estimated by extracting a self‐gating signal and applying focused navigation. Bulk motion was estimated by splitting the acquisition into sequential bins, reconstructing 3D volumes and applying rigid image registration. These combined motion estimates were used to correct k‐space. Self‐gating signals were compared to ground truth in simulations and an external sensor in utero. The cumulative position error (CPE) measured the accuracy of motion estimations relative to ground truth in simulations and relative sharpness measured the corresponding impact on image quality for both simulations and in utero data. An expert reviewer performed a blinded ranking of in utero images including uncorrected and corrected data.

**Results:**

Self‐gating signals correlated strongly with ground truth for simulations (*R* = 0.97 ± 0.01) and a external sensor for in utero data (*R* = 0.75 ± 0.23). CPE decreased significantly using ACROBATIC (uncorrected: 13.33[12.73–14.05], corrected: 2.20[1.80–2.61]). Relative image sharpness increased with ACROBATIC for both simulated (4.51[2.89–5.79]) and in utero data (1.12[0.77–1.44]) consistent with expert ranking where ACROBATIC images were given the best rank in the majority of cases.

**Conclusion:**

ACROBATIC enables motion correction in 3D radial fetal MRI. Correction of displacement due to maternal respiration and bulk motion results in improved image quality in simulations and in utero. Comparison to 2D frameworks are now warranted to establish the added diagnostic value of this approach.

## 1 Introduction

Fetal magnetic resonance imaging (MRI) plays an important role as a secondary modality during pregnancy following ultrasound [1]. In instances where acoustic shadowing, maternal obesity, or oligohydramnios limits diagnostic quality, the complementary use of MRI may improve prenatal diagnosis and delivery planning [2, 3]. To prevent artifacts and blurring caused by maternal respiratory motion and gross fetal motion, fetal MRI acquisitions are designed to minimize scan times [4]. At the same time, high spatial resolution is required to visualize the fetal anatomy, high temporal resolution is necessary to observe dynamic processes, and three‐dimensional (3D) coverage is needed to assess the developing fetal anatomy.

As the inherently prolonged scan time required for high resolution volumetric acquisitions increases the chances of fetal motion during the acquisition, a way to reject or correct for motion afflicted parts of the acquisition is required to achieve diagnostical useful image quality. In previous work, combinations of motion correction, scattered data interpolation, and super‐resolution algorithms [5, 6, 7], have successfully reconstructed 3D volumes from two‐dimensional (2D) image acquisitions. Still, 2D acquisitions may encounter limitations due to through‐plane motion and constrained spatial resolution in the slice selection direction. In contrast, 3D imaging [8, 9] may simplify scan planning and enable the simultaneous acquisition of multiple fetal organs of interest. Furthermore, isotropic voxels enable the retrospective selection of arbitrary imaging planes without discontinuities between slices which is important in assessing a number of congenital conditions. To account for respiratory motion in 3D acquisitions, respiratory navigators have been proposed [10], while fetal bulk motion has been addressed with outlier rejection [8] in radial sequences and translational motion correction in Cartesian sequences [11]. However, the use of navigators and data rejection impacts scanning efficiency.

The goal of this work is to develop a method for automatic correction of respiratory and bulk patient motion (ACROBATIC) in 3D radial fetal MRI. ACROBATIC estimates displacement of the fetal anatomy due to both maternal respiration and fetal bulk motion, using a combination of focused navigation [12] and image registration [6] enabling full use of the acquired data. ACROBATIC is developed and tested first in numerical simulations with comparisons to ground‐truth motion and its feasibility is demonstrated in a cohort of fetal subjects scanned in utero. This work tests the hypothesis that correcting motion with ACROBATIC enhances the quality of 3D radial fetal MRI.

## 2 Methods

### 2.1 ACROBATIC Framework

ACROBATIC is designed to correct rigid motion in free‐running 3D spiral phyllotaxis radial data [13, 14]. The large field‐of‐view (FOV) provided by this sequence covers the fetal anatomy and therefore, as a preparation step, a specific location for motion correction (i.e., brain, heart) can be chosen as follows. First, a gridded reconstruction of the entire FOV is performed using the non‐uniform fast‐Fourier transform (NUFFT). Then, spatial coordinates centered on the anatomy of interest are manually identified using a graphical user interface and a phase shift is applied to the original k‐space data such that all subsequent motion correction and reconstruction steps are performed with images centered on the user‐defined location. Note that this manual step may be omitted if the data are already acquired with the anatomy of interest centered in the FOV.

**FIGURE 1:**
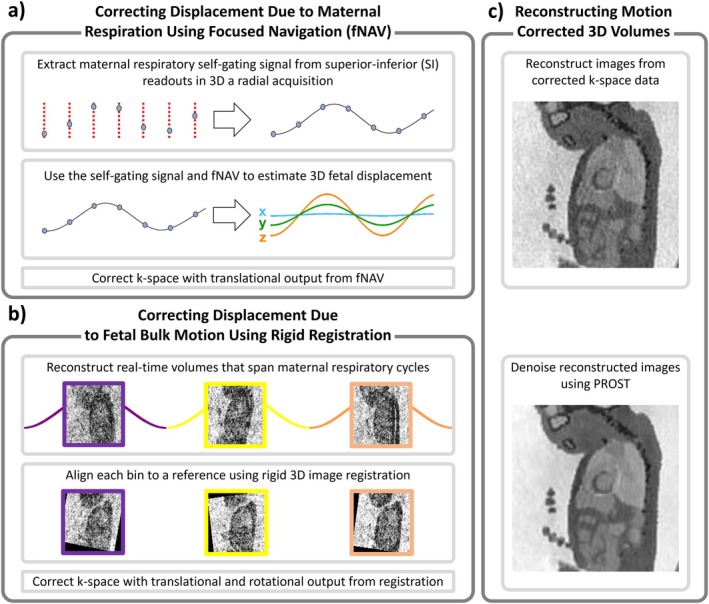
Schematic overview of the ACROBATIC motion correction framework with its individual steps: (a) Correcting displacement due to maternal respiration using focused navigation, (b) Correcting Displacement Due to Fetal Bulk Motion Using Rigid Registration, (c) Reconstructing Motion Corrected 3D Volumes.

The proposed ACROBATIC framework (Figure 1) is comprised of three components that are run in a single code without manual intervention: (1) the correction of fetal displacement due to maternal respiration, (2) the correction of fetal bulk motion, (3) and the reconstruction of motion corrected 3D volumes.

#### 2.1.1 Correcting Displacement due to Maternal Respiration Using Focused Navigation (fNAV)

The k‐space trajectory used in this work contains a repeated radial readout oriented along the superior–inferior (SI) direction enabling the extraction of a respiratory self‐gating signal using principal component analysis and bandpass filtering [14] (Figure 1a). Building on a method called focused navigation (fNAV) [12, 15], we posit that displacement of the fetus due to maternal respiration can be modeled by the multiplication of the self‐gating signal with three coefficients representing the maximum displacement along each spatial dimension. The values for these coefficients are initially set to 0 and then determined by an iterative optimization problem [16]. For each iteration, displacement is calculated by multiplying the self‐gating signal with the fNAV coefficients, applying a corresponding phase shift to the acquired k‐space data, and reconstructing a preliminary 3D image. The entropy of the gradient image is calculated over a region of interest (ROI) and the coefficients are updated according to a gradient descent approach that minimizes the cost function defined by the entropy measure. The optimized coefficients are then used to calculate a corresponding phase shift that is applied to the original k‐space data to correct for the effects of maternal respiratory motion.

#### 2.1.2 Correcting Displacement due to Bulk Motion Using Rigid Registration

Following respiratory motion correction, we exploit the flexibility of the 3D radial sampling pattern to perform retrospective “real‐time” reconstructions of the data that can be used to estimate bulk motion of the fetus via image registration. Here, the term real‐time refers to binning the k‐space data into sequential timepoints and then reconstructing dynamic 3D images by applying the NUFFT to each bin. To decouple displacement due to maternal respiratory motion from displacement due to fetal bulk motion, the length of these temporal bins was chosen such that each bin contains one or more complete maternal breathing cycles as measured from the self‐gating signals. This ensures that intra‐bin motion is primarily influenced by maternal respiration and is corrected by fNAV as described in the previous step.

Each real‐time bin is co‐registered to a reference using 3D rigid registration that maximizes mutual information [17, 18] with an evolutionary optimizer [19]. The reference (Figure 1b) is automatically chosen as the bin that shares the highest mutual information with all other bins [20]. The resulting 3D rigid transformation output is used to correct the acquired k‐space data in two steps. First, the coordinates of the acquired k‐space trajectory are transformed using the rotation matrix output from the rigid transformation. Second, the translational component of the registration output is used to calculate a corresponding phase shift that is applied to the k‐space data to correct for the effects of fetal bulk motion.

#### 2.1.3 Reconstructing Motion Corrected 3D Volumes

In the previous two steps, phase shifts have been applied first to the original k‐space data to correct displacement due to respiratory motion, and then to correct for bulk motion. Final image reconstructions (Figure 1c) are then performed by applying a NUFFT, that contains the corrected trajectory described in the previous step, to the corrected k‐space data thus producing motion corrected 3D volumes. Denoising of these volumes is performed using low‐rank patch‐based PROST [21].

#### 2.1.4 Tuning Parameters

For each component of the ACROBATIC framework, tuning parameters were explored in parameter studies (see Supporting Information). As the anatomy of interest is centered within the FOV from the acquisition or using the aforementioned preparation step, no manual steps are required. The only tuning parameter for fNAV is the size of the ROI which was fixed to a cubical volume of (132 mm)^3^ for simulated data and (60 mm)^3^ for in utero data (see Supporting Information). Two tuning parameters for bulk motion correction are needed. First, the temporal width of real‐time bins was determined such that the minimal number of radial spokes per bin was 1500 spokes, resulting in approximately 30–40 bins per acquisition with a retrospective real‐time temporal resolution of ∼8 s. Second, the ROI used for image registration was fixed to a cubical volume of (132 mm)^3^ for simulated data and (60 mm)^3^ for in utero data. There are four tuning parameters for PROST denoising. These include the patch size of 4 x 4 × 4 voxels, a search window radius of 20, a number of similar selected patches of 20, and a spatial regularization weight of 0.1. These parameters were kept fixed based on previous work with the exception of the spatial regularization weight which was explored and optimized in the aforementioned parameter studies provided in the Supporting Information.

### 2.2 Numerical Simulation Model

**FIGURE 2:**
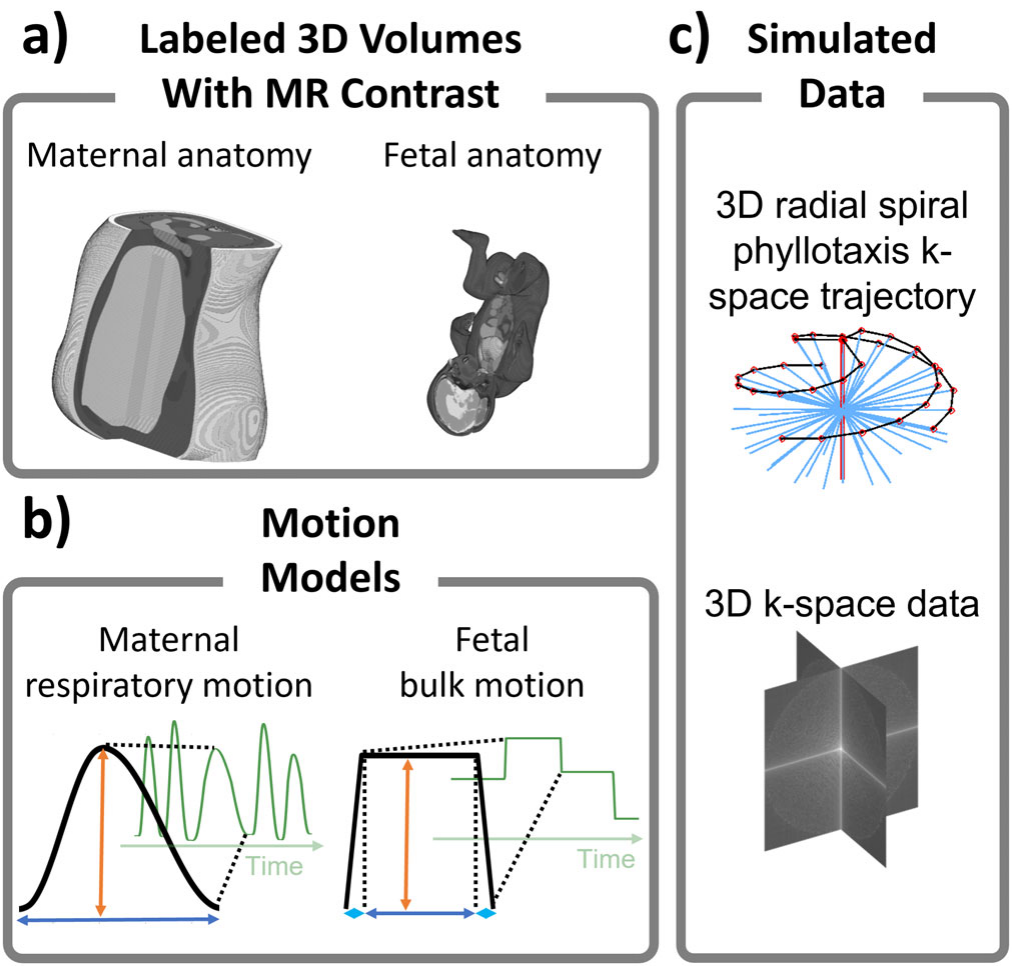
Schematic overview of the numerical simulation model: (a) Labeled 3D Maternal and 3D fetal anatomies are generated with MR contrast. (b) Motion models for maternal respiratory motion and fetal bulk motion are created with respective parametrized motion curves (orange/blue arrows). (c) The data acquisition is simulated with a 3D radial spiral Phyllotaxis trajectory to generate a 3D k‐space.

A numerical simulation model was created for validation in this work based on the previously described MRXCAT and fetal XCMR approaches for 2D imaging [22, 23] (Figure 2). Here we incorporate models for 3D rigid motion of the fetus and enable simulation of 3D radial MR acquisitions. The simulation and reconstruction code, along with example data, will be made available: https://github.com/cwroy/Fetal‐XCMR.

#### 2.2.1 Data Generation

The simulation uses high‐resolution 3D arrays of maternal and fetal anatomy from the XCAT phantom [22, 23] (Figure 2a), generated across 50 time points to simulate maternal respiratory and fetal cardiac motion. Maternal motion includes rigid and non‐rigid components, for example, bladder and intestinal deformation, and amniotic sac translation (∼6 mm anterior–posterior, ∼3 mm superior–inferior). Fetal bulk motion is modeled independently but constrained by the amniotic sac. Labeled tissues (i.e., blood, muscle, bone, etc.) from the XCAT arrays were converted to MR contrast as follows. For each tissue, the corresponding MR signal was calculated using an analytical equation for bSSFP wherein the T1 and T2 relaxation values were taken from the literature, and sequence parameters are those described in the Numerical Simulation Data section below [22]. The fetal anatomy was transformed according to motion models for maternal respiration and fetal bulk motion, with 3D translation and 3D rigid motion, respectively (Figure 2b). A 3D radial trajectory was simulated to sample maternal and fetal 3D anatomies according to their respective motion states with a NUFFT to create k‐space data (Figure 2c). Acquisition parameters were chosen to replicate those used for in utero scanning as described in the In Utero Data section below. To add variability, the fetal anatomy with an original biparietal diameter of ∼90 mm, simulating late gestation (∼37 weeks gestational age) [24], was scaled to a biparietal diameter of ∼67 mm, simulating mid‐late gestation (∼27 weeks gestational age) [24], both for cephalic and breech positions. This was done through scaling and rotation of the fetal anatomy which does not fully replicate physiological conditions but provides an otherwise unavailable ground truth reference and controlled means to systematically assess the robustness of the proposed motion correction framework across varying extents of fetal motion and anatomical scales.

#### 2.2.2 Respiratory Motion Model

The simulated maternal respiratory motion curve (Figure 2b) was derived from a reference signal obtained in vivo. Breath‐to‐breath intervals were modified in amplitude and period to introduce variability. This enabled programming fetal translation in three dimensions via scaled respiratory curves (amplitude < 7.5 mm), consistent with prior studies [6, 7, 20].

#### 2.2.3 Fetal Bulk Motion Model

Fetal bulk motion was modeled as resting and transition periods, based on fetal ultrasound actograms [25, 26]. Late gestation featured fewer, longer resting phases (2–5 per acquisition), while mid‐late gestation showed increased motion frequency (2–10 per acquisition). Each resting period was defined by a duration and a fetal position, given by pairs of 3D translation and rotation of the fetal anatomy. Motion ranges between resting periods were informed by prior studies [6, 7, 20], and provided an initial test‐range for AROBATIC. These included: late gestation (±7.5 mm, ±5°) and mid‐late gestation (±15 mm, ±10°). Simulated datasets included low (±1.5 mm/±1° and ±3 mm/±2°) and high (±7.5 mm/±5° and ±15 mm/±10°) motion levels for both gestational ages, with fetuses simulated in cephalic and breech positions. Motion periods were linear transitions between resting positions. The length and distribution of the resting and motion periods were parameterized (Figure 2b) and then quasi‐randomly distributed throughout the simulated acquisition. The amplitudes of this parameterized fetal bulk motion model were then scaled for both translation and rotation.

### 2.3 Study Data

#### 2.3.1 Numerical Simulation Data

Two hundred fetal datasets were simulated to evaluate ACROBATIC. MR sequence parameters were chosen to closely match those used in utero. Key parameters included a 3D radial spiral Phyllotaxis k‐space trajectory with 22 readouts per radial interleave and a total of 3977 interleaves [13]. We simulated bSSFP contrast with TE/TR: 1.44/2.88 ms, isotropic spatial resolution: (1.5 mm)^3^, and FOV: (288 mm)^3^ for a total simulated acquisition time of 4 min 12 s.

#### 2.3.2 In Utero Data

Twelve pregnant women (32 ± 2 weeks gestational age) were scanned to test the feasibility of ACROBATIC applied in utero. Each subject underwent clinically indicated MRI and consented to this institutional review board‐approved study. As an additional research scan, free‐running 3D radial bSSFP data with a spiral phyllotaxis k‐space trajectory were acquired on 1.5 T scanners (MAGNETOM Aera/Sola, Siemens Healthineers, Erlangen, Germany). Parameters included TE/TR: 1.44/2.87–2.02/4.07 ms, resolution: (1.0–1.5 mm)^3^, FOV: (256–320 mm)^3^, and acquisition time: 4:11–6:00 min. Variations were due to the size of the maternal anatomy and the gestational age of the fetus. The entire fetal anatomy was captured within the fully sampled FOV due to the 2× oversampling along the readout direction. In nine cases, a BioMatrix sensor (Siemens Healthineers, Erlangen, Germany) recorded a maternal respiratory signal during the acquisition.

### 2.4 Evaluation Metrics

The components of ACROBATIC (respiratory and bulk motion correction) were validated by a series of metrics that assess the ability to estimate and correct motion as well as its impact on the quality of reconstructed images. To understand the impact of each step, we evaluated these metrics in four types of image reconstructions: (1) without applying any motion correction (uncorrected), (2) correcting only for displacement due to maternal respiration (respiratory motion correction), (3) correcting only for fetal bulk motion (bulk motion correction) and (4) using the full ACROBATIC framework (respiratory and bulk motion correction). For a fair comparison, all image reconstructions were denoised using PROST. For both simulated and in utero data all motion correction and image reconstruction steps were performed and evaluated twice. Once with the volume manually centered on the fetal heart and once centered on the fetal brain. The results from the metrics are then reported as the average of the two anatomical locations.

#### 2.4.1 Respiratory Self‐Gating Signals

Self‐gating signals were compared to ground truth respiratory motion for all simulation data using the Pearson correlation. In the absence of ground truth in utero, the maternal respiratory signal from the BioMatrix sensor was also compared to the self‐gating signal using Pearson correlation to provide external validation.

#### 2.4.2 Motion Estimates

To validate our estimates of fetal displacement due to maternal respiration and fetal bulk motion using ACROBATIC we defined the cumulative position error (CPE). This provides a comparison between the estimated motion using ACROBATIC and the ground truth motion provided by the numerical simulation model. To calculate the CPE first consider a cubical 3D volume V_in_ with its eight 3D vertex coordinate vectors a_i_(t) ∈ ${\mathbb{R}}^{3}$, with i ∈ {1, 2, …, 8}, for a time point (t) during the acquisition, translated and rotated to a position given by the ground truth 6D input‐motion. Similarly, consider a Volume V_out_ with its vertex coordinate vectors a'_i_(t), translated and rotated to a position given by the estimated motion from fNAV, image registration, or both. By calculating the Euclidean norm of the vector differences ∣∣a_i_(t)‐a'_i_(t)∣∣, the cumulative position error in units of voxels can be calculated by averaging over all vertex coordinates and all acquisition time points.

(1) $$\mathrm{CPE}=\frac{\sum_{t}^{N_{t}}\sum_{i}^{8}\left\|a_{i}\left(t\right)-a_{i}^{\prime }\left(t\right)\right\|}{8N_{t}\;}$$

With N_t_ = 10 000 being the number of time points evenly spaced across the acquisition. By defining both volumes V_in_ and V_out_ to match the ROI dimensions used in the reconstruction framework for fNAV and registration, the CPE quantifies how accurately fetal motion is estimated within these cubical volumes. For purely translational motion, vertex displacements computed by the CPE are similar to voxel displacements within the volume. In contrast, for rotational motion, vertex displacements capture the maximal voxel displacements, thereby providing an upper bound on the displacement error. Although the fetal anatomy is not cubic in shape, computing the CPE within bounding‐box volumes enables a practical approximation of the motion estimation error, with a systematic residual discrepancy reflecting the deviation between the bounding box and the true anatomical boundaries. The CPE is calculated for both levels of bulk motion and both simulated gestational ages. The CPE values calculated for individual steps of the ACROBATIC framework are compared using a Wilcoxon signed‐rank test. CPE values are reported as median and interquartile range [Q1–Q3].

#### 2.4.3 Impact of Motion Correction on Image Quality

To evaluate image quality across individual steps of the ACROBATIC framework in all 200 simulated and 11 in utero datasets, we applied a metric for relative sharpness between uncorrected and corrected reconstructions [20]. Briefly, 3D Gaussian blur with progressively larger kernels was applied to motion‐corrected images and the root mean‐squared error (RMSE) was calculated between these blurred images and their uncorrected counterparts. The relative sharpness is then defined as the kernel in units of voxels, that minimizes the RMSE. A higher value indicates a bigger difference between uncorrected and motion corrected reconstructions and thus higher sharpness of the motion corrected reconstruction. The relative sharpness was assessed for all individual steps of the ACROBATIC framework for both simulated and in utero data. For simulated data this included analysis of each motion level and gestational age. Differences in relative sharpness across reconstruction steps were assessed using the Wilcoxon signed‐rank test. Values are reported as median and interquartile range [Q1–Q3].

Additionally, an expert in fetal MRI, blinded to reconstruction details, qualitatively evaluated all in utero datasets across locations (heart and brain) and ACROBATIC steps (uncorrected, respiratory‐corrected, bulk‐corrected, combined respiratory and bulk motion corrected). For each dataset, the reconstruction order was randomized, and images were ranked from best (1) to worst (4).

### 2.5 Computation Time

All reconstruction and evaluation steps were performed in MATLAB (MathWorks—Natick, MA, USA), on a workstation equipped with a 24‐core AMD Ryzen Threadripper PRO 7965WX CPU (AMD, Santa Clara, CA, USA), 1.0 TB of RAM and a NVIDIA RTX 6000 Ada Generation GPU (Nvidia, Santa Clara, CA, USA). The computational time for individual steps was recorded for subsequent comparisons.

## 3 Results

Table 1 summarizes the performance of ACROBATIC in terms of estimating motion and the impact on image quality for simulated and in utero data.

**TABLE 1:** An overview of all quantitative results.

(a) Respiratory motion extraction		
	Simulations	In utero
Pearson correlation	0.97 ± 0.01	0.75 ± 0.23

(b) Cumulative position error				
	Uncorrected	Respiratory motion corrected	Bulk motion corrected	Respiratory and bulk motion corrected
Late gestation, low motion	1.89 [1.79–2.02]	1.50 [1.37–1.65]	1.53 [1.41–1.84]	1.02 [0.74–1.37]
Mid‐late gestation, low motion	3.09 [2.83–3.25]	2.86 [2.63–2.99]	1.56 [1.39–1.75]	0.99 [0.75–1.26]
Late gestation, high motion	7.10 [6.60–7.44]	7.00 [6.51–7.36]	1.72 [1.52–2.01]	1.19 [0.95–1.56]
Mid‐late gestation, high motion	13.33 [12.73–14.05]	13.27 [12.69–14.00]	2.55 [2.23–2.96]	2.20 [1.80–2.61]

(c) Relative Sharpness				
	Respiratory motion corrected	Bulk motion corrected	Respiratory and bulk motion corrected	Ground truth
Late gestation, low motion	0.61 [0.56–0.74]	0.50 [0.47–0.55]	0.76 [0.65–0.84]	0.78 [0.68–0.87]
Mid‐late gestation, low motion	0.64 [0.56–0.72]	0.90 [0.78–1.14]	1.06 [0.96–1.31]	1.09 [1.01–1.30]
Late gestation, high motion	0.67 [0.59–0.78]	1.82 [1.17–2.51]	2.00 [1.33–2.51]	2.01 [1.50–2.62]
Mid‐late gestation, high motion	0.72 [0.61–0.82]	4.51 [2.66–5.77]	4.51 [2.89–5.79]	5.01 [3.01–5.96]
In utero	0.50 [0.45–0.59]	1.07 [0.70–1.33]	1.12 [0.77–1.44]	/

### 3.1 Respiratory Self‐Gating Signals

Self‐gating signals from (*N* = 200) simulated acquisitions showed a strong correlation (*R* = 0.97 ± 0.01) with the ground truth respiratory signal. For nine in utero datasets, self‐gating signals also showed a strong correlation (*R* = 0.75 ± 0.23) with the external respiration signal.

### 3.2 Motion Estimates

**FIGURE 3:**
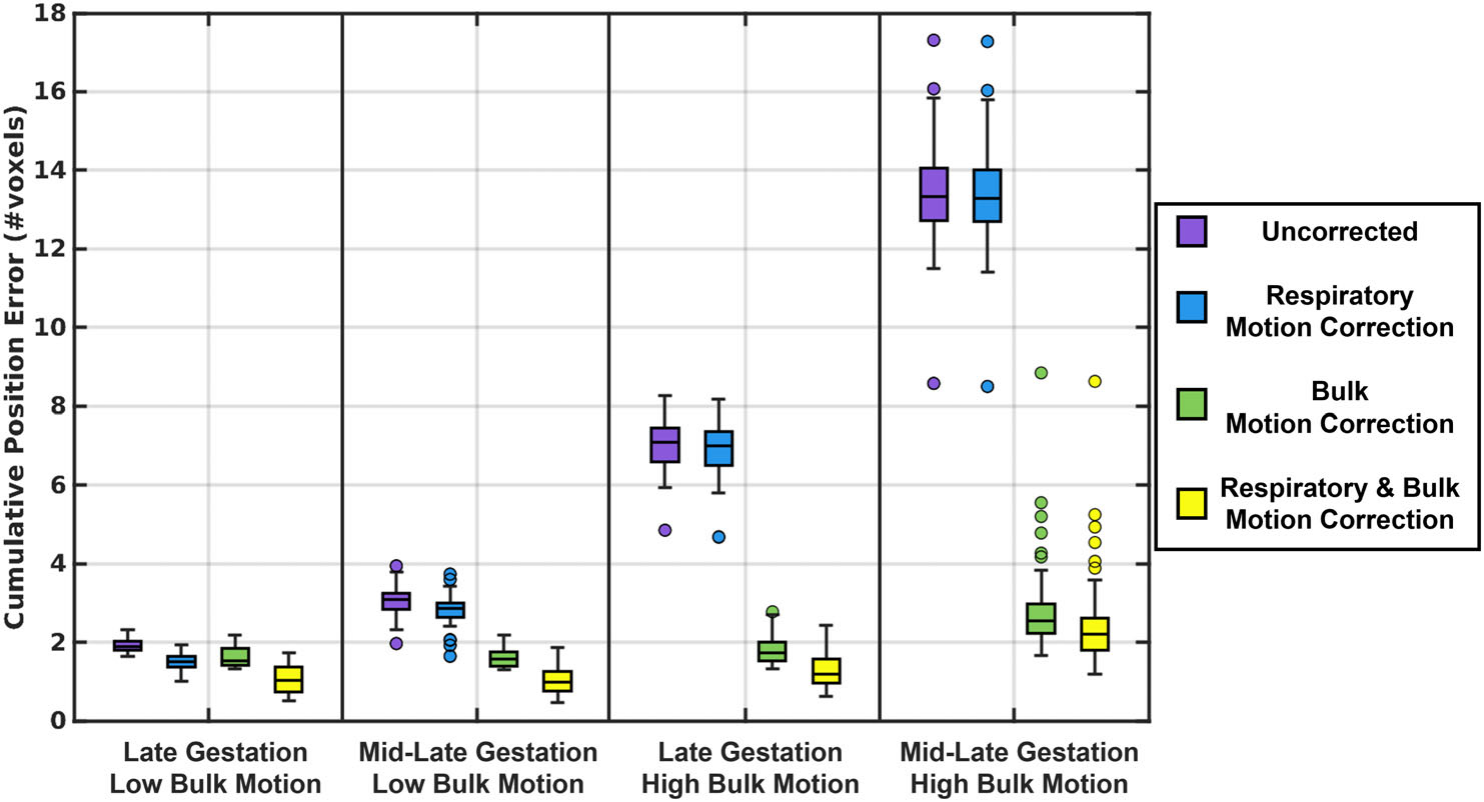
Cumulative position error (CPE) for all 200 simulated acquisitions and two respective ROIs (brain, heart), divided into the different bulk motion levels (low, high), the simulated gestational ages (late, mid‐late) and in dependence of the proposed motion correction steps: Uncorrected, respiratory motion correction, bulk motion correction, and combined respiratory and bulk motion correction. For each gestational age and motion level, all CPE values differed significantly across motion correction steps (*p* < 0.05).

For data simulating late gestation and low bulk motion (Figure 3), uncorrected images had a low positional error (CPE = 1.89 [1.79–2.02]), which decreased following respiratory motion correction (CPE = 1.50 [1.37–1.65]) and bulk motion correction (CPE = 1.53 [1.41–1.84]), with the lowest error observed when correcting for both sources of motion (CPE = 1.02 [0.74–1.37]). For mid‐late gestation and low motion, uncorrected data yielded higher error (CPE = 3.09 [2.83–3.25]), which was reduced after respiratory motion correction (CPE = 2.86 [2.63–2.99]) and bulk motion correction (CPE = 1.56 [1.39–1.75]) and minimized when correcting both (CPE = 0.99 [0.75–1.26]). For late gestation and high motion, uncorrected reconstructions showed increased error (CPE = 7.10 [6.60–7.44]), with small improvement after respiratory motion correction (CPE = 7.00 [6.51–7.36]), but less error following bulk motion correction (CPE = 1.72 [1.52–2.01]) and further decreased error when correcting both (CPE = 1.19 [0.95–1.56]). For mid‐late gestation and high motion, uncorrected images exhibited the highest error (CPE = 13.33 [12.73–14.05]), which decreased slightly after respiratory motion correction (CPE = 13.27 [12.69–14.00]) and more substantially after bulk motion correction (CPE = 2.55 [2.23–2.96]), with the lowest error again achieved through correcting both (CPE = 2.20 [1.80–2.61]). For each gestational age and motion level, all CPE values differed significantly across reconstruction steps (*p* < 0.05).

### 3.3 Impact of Motion Correction on Image Quality

**FIGURE 4:**
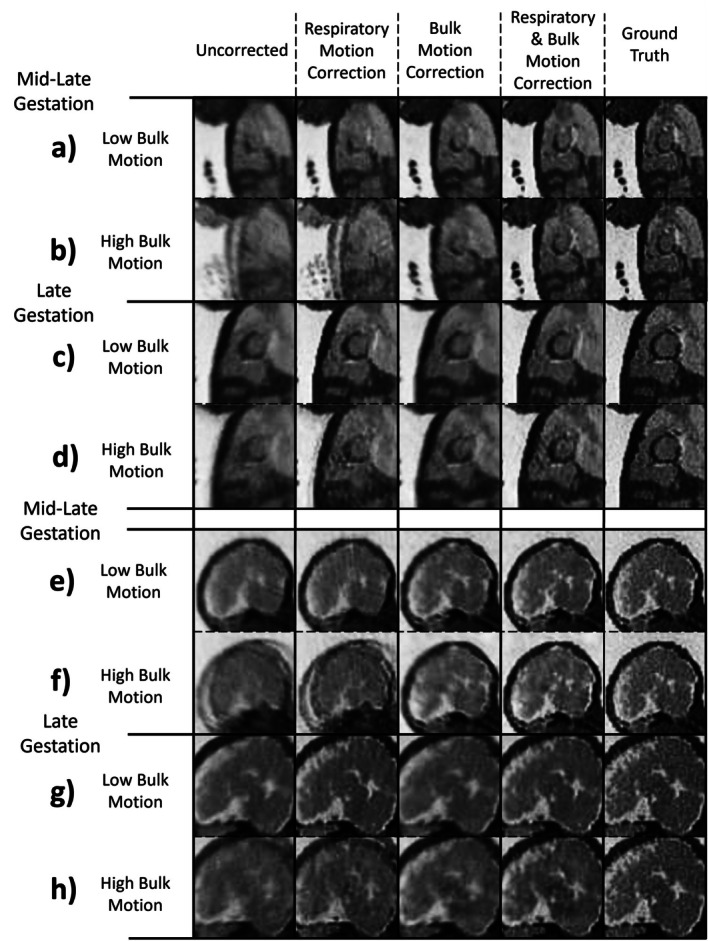
Exemplary static reconstructions of eight simulated acquisitions, centered on the heart (a–d) and the brain (e–h) and divided into 2 bulk motion levels (low: a,c,e,g; high: b,d,f,h) and into late‐mid gestation (a,b,d,e) and late gestation (c,d,g,h) in sagittal view with all motion correction steps: Uncorrected, respiratory motion correction, bulk motion correction, and combined respiratory and bulk motion correction. The simulated ground truth (without motion) is also visualized. PROST denoising is applied to all the shown reconstructions.

The measurements of CPE are complemented by the visualization of reconstructed simulation data with anatomical locations centered on the heart (Figure 4a–d), and the brain (Figure 4e–h). For data simulating late gestation and low bulk motion (Figure 4c,h), respiratory motion correction alone improves image quality, recovering most fine anatomical details relative to the ground truth. For mid‐late gestation and low motion (Figure 4a,f), respiratory motion correction yields modest improvement in perceived image quality, whereas correcting both sources of motion markedly enhances image quality and restores fine anatomical detail. In contrast, for both gestational ages and high motion (Figure 4b,d,f,h), image quality comparable to the ground truth is only achieved when both sources of motion are corrected.

**FIGURE 5:**
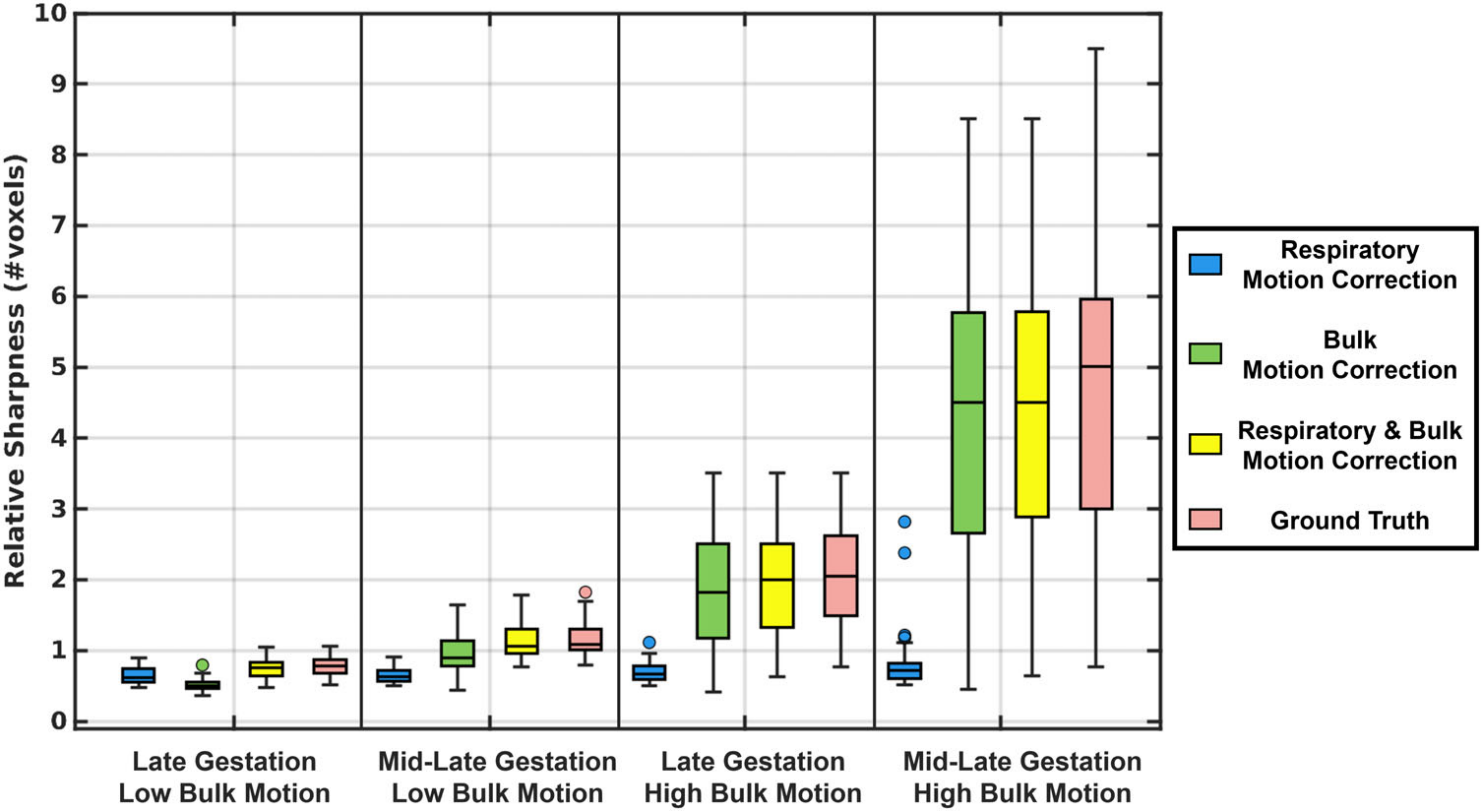
Relative sharpness analysis of the respective ACROBATIC motion correction steps (Respiratory motion correction, bulk motion correction, and combined respiratory and bulk motion correction) for all 200 simulated acquisitions, divided into different bulk motion levels (low, high), and the gestational age (mid‐late, late). For each gestational age and motion level, all relative sharpness values differed significantly across motion correction steps (*p* < 0.05).

These results are further complemented by relative sharpness measurements (Figure 5). For data simulating late gestation with low bulk motion, respiratory motion correction improved sharpness (0.61 [0.56–0.74]) more than bulk motion correction (0.50 [0.47–0.55]), while correcting both (0.76 [0.65–0.84]) yielded values approaching ground truth (0.78 [0.68–0.87]). Conversely, for mid‐late gestation and low motion, respiratory motion correction improved sharpness (0.64 [0.56–0.72]), less than bulk motion correction (0.90 [0.78–1.14]), and again, correcting both (1.06 [0.96–1.31]), yielded values slightly below the ground truth (1.09 [1.01–1.30]). For late gestation and high motion, respiratory motion correction (0.67 [0.59–0.78]) was also less effective than bulk motion correction (1.82 [1.17–2.51]), while correcting both (2.00 [1.33–2.51]) matched ground truth sharpness (2.01 [1.50–2.62]). Finally, in mid‐late gestation with high bulk motion, respiratory motion correction improved sharpness (0.72 [0.61–0.82]), much less than bulk motion correction (4.51 [2.66–5.77]), and combined correction (4.51 [2.89–5.79]), closely aligning with the ground truth (5.01 [3.01–5.96]). Across all gestational ages and bulk motion levels, image sharpness differed significantly between individual reconstruction steps (*p* < 0.05).

**FIGURE 6:**
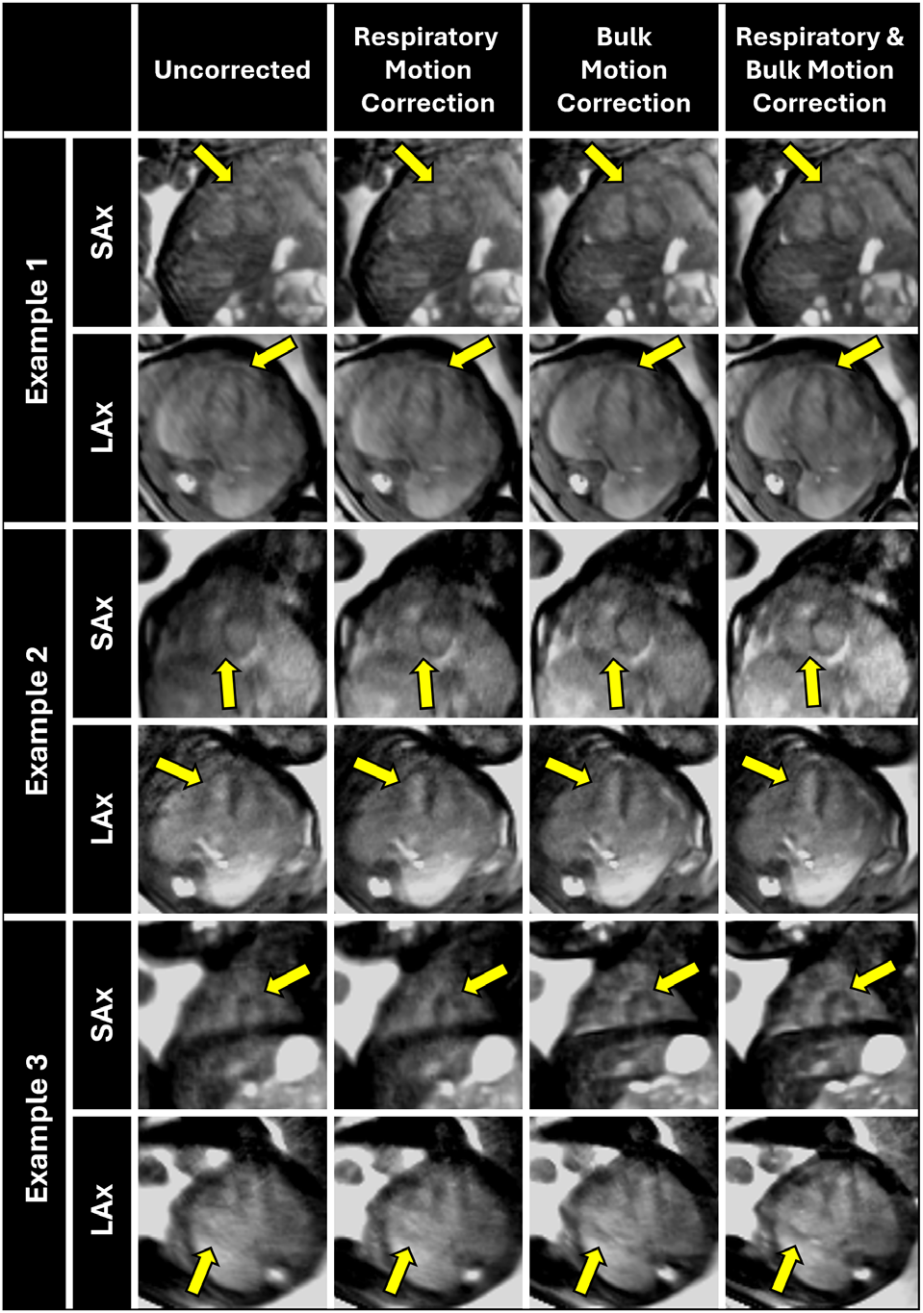
Exemplary static reconstructions of three in utero acquisitions centered on the fetal heart in short axis (SAx) and long‐axis (LAx) views with all motion correction steps: Uncorrected, respiratory motion correction, bulk motion correction, and combined respiratory and bulk motion correction. PROST denoising is applied to all the shown reconstructions. The yellow arrows mark the myocardium for the respective motion correction steps.

**FIGURE 7:**
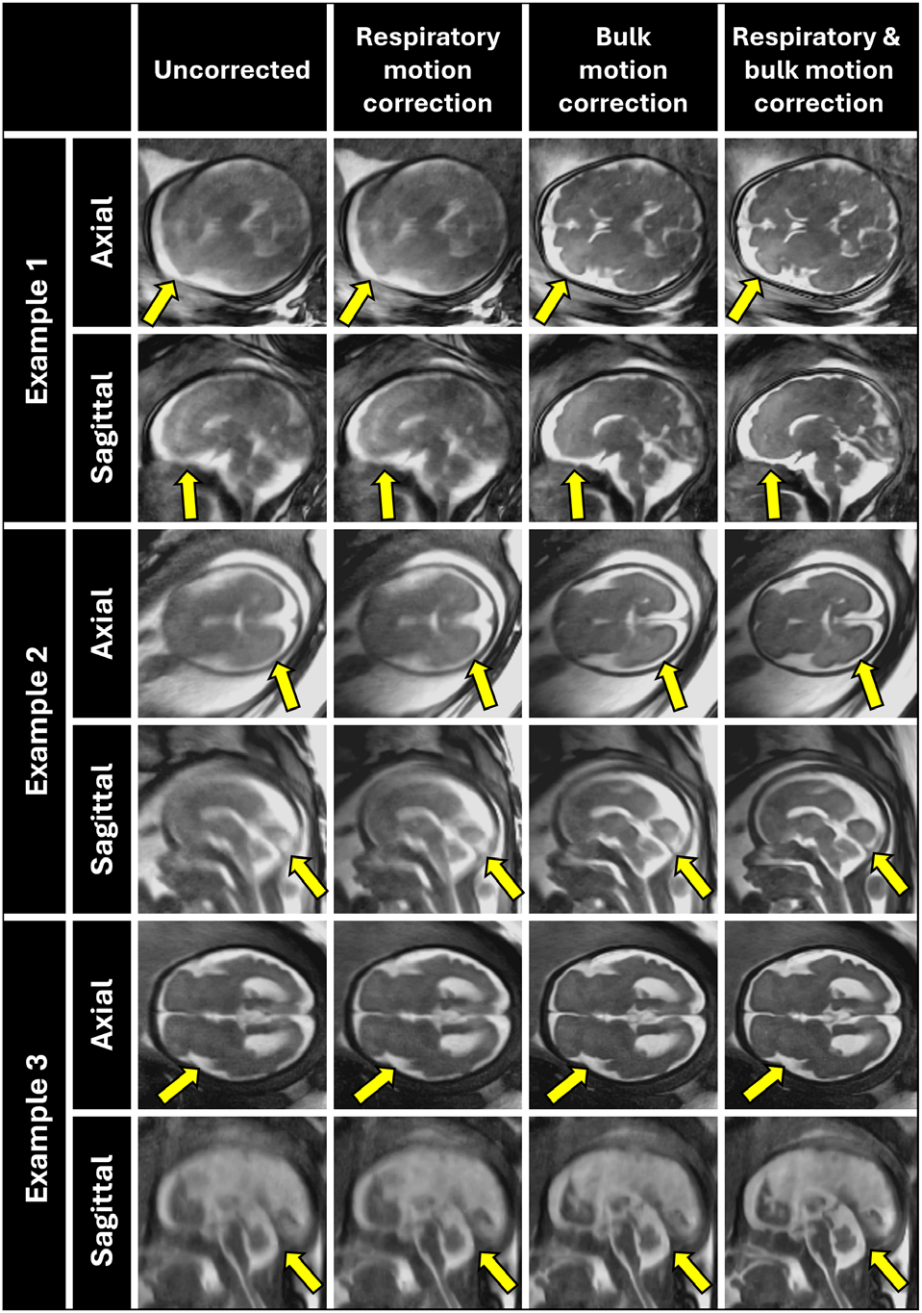
Exemplary static reconstructions of three in utero acquisitions centered on the brain in coronal, sagittal, and axial views with all motion correction steps: Uncorrected, respiratory motion correction, bulk motion correction, and combined respiratory and bulk motion correction. PROST denoising is applied to all the shown reconstructions. The yellow arrows highlight white‐gray matter interfaces for the respective motion correction steps.

For reconstructions centered on the heart of in utero data, bulk motion correction improved contrast and sharpness at the blood–myocardium interface (Figure 6, yellow arrows), whereas respiratory motion correction alone yielded subtler enhancement. For reconstructions centered on the brain of in utero data, sharpened gray–white matter interfaces are present (Figure 7, yellow arrows) following combined respiratory and bulk motion correction.

**FIGURE 8:**
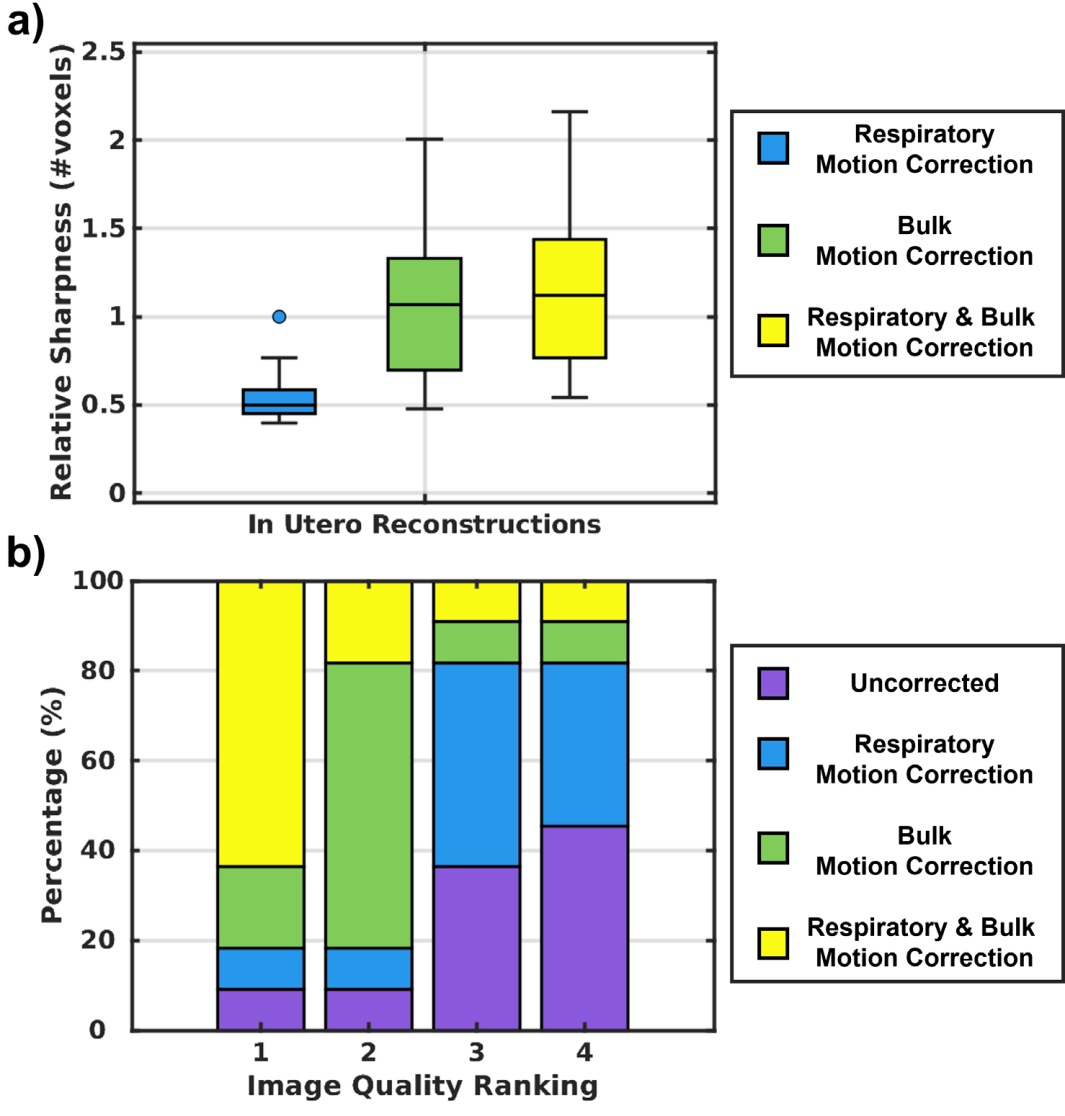
(a) Relative sharpness analysis of the respective ACROBATIC motion correction steps (Respiratory motion correction, bulk motion correction, and combined respiratory and bulk motion correction) for all in utero data. For each gestational age and motion level, all relative sharpness values differed significantly across motion correction steps (*p* < 0.05). (b) Expert ranking of in utero reconstructions. For each dataset, reconstructions of all motion correction steps (uncorrected, respiratory motion correction, bulk motion correction, and combined respiratory and bulk motion correction) were shown to an expert in random order and ranked from best to worst.

These results are further complemented by the measurements of relative sharpness in the 11 in utero datasets (Figure 8a). Respiratory motion correction alone yielded lower sharpness (0.50 [0.45–0.59]) than bulk motion correction (1.07 [0.70–1.33]), while correcting both further improved sharpness (1.12 [0.77–1.44]). Relative sharpness differences between individual motion correction steps were statistically significant (*p* < 0.05). Expert image rankings (Figure 8b) further substantiate the results, showing that uncorrected data were most frequently rated the worst (45%), whereas correcting displacement due to maternal respiration and fetal bulk motion was rated best (64%) in the majority of cases. The uncorrected images were only ranked best in 9% of cases.

### 3.4 Computation Time

The total reconstruction times for the simulated data were 9 min 55 s ±1 min 24 s, including 1 min 23 s ±7 s for respiratory motion correction, 7 min 21 s ±1 min 19 s for bulk motion correction, and 24 s ±2 s for final reconstruction with denoising. For in utero data, the reconstruction time was 8 min 51 s ±1 min 18 s, including 1 min 42 s ±17 s for respiratory motion correction, 4 min 9 s ±49 s for bulk motion correction, and 27 s ±3 s for final reconstruction with denoising.

## 4 Discussion

In this work, we developed and validated ACROBATIC to estimate and correct displacement of the fetus due to maternal respiration and bulk motion in 3D radial MRI acquisitions. We successfully tested our hypothesis that ACROBATIC improves 3D images of the fetal heart and brain as assessed both by quantitative and qualitative metrics. This approach enables coverage of the entire fetus within the acquired FOV and provides retrospective motion correction and visualization of multiple anatomical locations from the same acquisition.

### 4.1 Numerical Simulation Model

A numerical simulation was developed for this work to provide an otherwise unavailable ground truth to test the components of the ACROBATIC framework. Simulations have been shown [7, 20, 23] to enhance the development of fetal MRI methods, providing valuable translational knowledge for reconstructing data acquired in utero. Nevertheless, an analytical signal model was used to generate MR contrast rather than a Bloch or EPG simulation and therefore spin histories, eddy currents, flow effects, and off‐resonances were not considered. Furthermore, different gestational ages were simulated by scaling the fetal model and not considering differences in tissue properties which change rapidly during fetal growth. Simulated fetal bulk motion may also deviate from what is found in utero. While we based our motion model on published ranges, it could be enhanced with non‐rigid and intra‐bin dynamics (e.g., startles, fetal breathing [25, 27]). Improvements to the simulation, especially in the signal model, may help bridge the gap between simulations and in utero acquisitions to better enable studies that consider alternative sequences including comparisons between 2D and 3D acquisitions. The numerical simulation could also be complemented by in vitro tests using motion phantoms [28, 29] to further connect simulated results to real‐world scanning conditions. Nevertheless, to estimate motion and evaluate its impact on image quality, the current numerical simulation model provided a valuable tool to develop ACROBATIC and test the hypothesis of this work.

#### 4.1.1 Respiratory Self‐Gating Signals

Self‐gating plays an important role in the respiratory motion correction step of ACROBATIC. Strong correlations [30] were observed between self‐gating signals and their ground truth simulated counterparts as well with an external sensor in utero. However, the correlation was notably higher for simulated data. This may reflect deficiencies in the realism of simulated signal model, in particular the lack of eddy current effects that are known to impact self‐gating signals [14]. This may also indicate a difference in how the self‐gating and external sensor signals decouple fluctuations caused by respiration and bulk motion. Despite these limitations, the external sensor provided a reference to demonstrate the feasibility of extracting a maternal respiratory self‐gating signal from data acquired in utero. Further improvement could include a spatially localized respiration signal through a method like MRI compatible Doppler ultrasound [8, 9, 31]. Alternatively, an MR‐based respiratory navigator could be used for a spatially localized signal but would require interruptions to the MR sequence, impacting both efficiency and ability to maintain a steady‐state signal.

#### 4.1.2 Motion Estimates

Estimations of displacements due to maternal respiratory motion and fetal bulk motion were consistently achieved by ACROBATIC as demonstrated by the CPE values (Figure 3) and provided a clear impact on image quality for both simulated and in utero data. Still, the motion estimation process was performed in two parts resulting in sequential phase shifts applied to the original k‐space data. To investigate potential interference of these two steps, evaluation metrics were calculated for the individual components of ACROBATIC. The end results show that the combination of respiratory motion correction and bulk motion correction is required to produce the sharpest images, but improvements may be needed to decouple the impact of these two steps. In particular, if the current temporal resolution (∼ 8 s per bin) of the real‐time reconstructions could be improved (< 1 s per bin), displacement due to maternal respiration and fetal bulk motion may be estimated and corrected in a single step. This may also better capture rapid fetal movements and extend the use of ACROBATIC to earlier gestation. Achieving higher temporal resolutions may be possible with modified k‐space trajectories that provide better coverage per unit time, or by exploring advanced reconstruction methods for the real‐time images [32, 33].

#### 4.1.3 Impact of Motion Correction on Image Quality

The quality of uncorrected reconstructions consistently improved after application of the ACROBATIC framework. These results underscore the importance of addressing both motion sources in 3D radial fetal MRI. When considering images reconstructed from in utero data, the ACROBATIC framework appears to perform slightly better for the brain (Figure 7) than the heart (Figure 6), due to the predominantly rigid motion of the head and higher contrast achieved with the current bSSFP sequence. The head may also exhibit less intra‐bin motion compared to other fetal regions prone to sudden, irregular movements [25, 27, 34]. Additionally, movement of the fetal torso is more difficult to distinguish from independent movements of the limbs and static maternal anatomy. Separating these anatomical locations may be solved by automatic masking of fetal structures in the real‐time reconstructions, allowing the registration to focus solely on relevant anatomy [35, 36]. The quality of cardiac images in the current study is also limited by uncompensated cardiac motion. A natural extension of ACROBATIC would be the integration of cardiac gating [8, 9, 31], to enable 4D motion‐resolved imaging of fetal cardiac anatomy and blood flow.

#### 4.1.4 Computation Time

ACROBATIC reconstructions were performed offline and took ∼9 min for in utero data. The majority of the computational time comes from the bulk motion correction step wherein multiple 3D volumes are reconstructed using the NUFFT in sequential order. This step could be significantly accelerated by parallelizing the computation and implementing it inline for clinical use, leveraging emerging techniques that integrate third‐party reconstruction tools directly with scanner hardware [37, 38, 39].

#### 4.1.5 Limitations

In this work, a 3D radial bSSFP sequence with spiral phyllotaxis sampling was used. This included a repeated projection along the SI direction to extract self‐gating signals [14] which enabled the use of fNAV for respiratory motion correction [12]. The flexibility of this trajectory allowed for retrospective real‐time images that tracked fetal bulk motion. This sequence served as a proof‐of‐concept to demonstrate how multiple anatomical locations could be motion corrected and visualized. Nevertheless, to evaluate the diagnostic utility of this approach, improvements to the image contrast and residual non‐motion artifacts are needed. Compatible 3D radial methods could include unbalanced SSFP [40] for black blood imaging, T1 enhanced SSFP [41], or adding flow‐encoding [9, 42, 43] to create a phase contrast angiogram. The inclusion of a strategy for reducing the signal from maternal fat in the free‐running sequence [44, 45] may be particularly beneficial as it remains a strong source of streaking artifacts. Regardless of the potential improvements to the sequence, both the numerical simulation and ACROBATIC could be adapted to track and correct motion provided a signal for maternal respiration is available from the sequence or hardware [46, 47] and a flexible k‐space trajectory is used which may alternatively consider Cartesian [21, 48, 49], other radial approaches [8, 50], or spiral cones [32, 51].

A significant limitation in the current study was the lack of 2D reference images for comparison with the motion corrected 3D images of the fetal heart and brain. In particular, breath‐held 2D CINE images are needed to understand the relative value of the 3D cardiac images and single‐shot T1 or T2 weighted images are needed to evaluate the brain. In the current work, the temporal resolution of a given bin (∼8 s) used to track fetal bulk motion is significantly lower than for previously published 2D methods that similarly used retrospective real‐time reconstructions [20, 52] to track motion with high temporal resolution (∼50 ms). However, the use of fNAV for intra‐bin correction and the ability to truly track the fetus in 3D may balance this limitation. Previous 2D methods have used an external gating device [8] and also been published with self‐gating solutions [20, 53, 54] that enable 2D CINE imaging and assessment of quantitative cardiac function. To compare function measurements with the current 3D approach, a fetal cardiac self‐gating method for 3D acquisitions would need to be developed or an external gating device would be needed [8, 9, 31]. Furthermore, the current 3D sequence does not benefit from inflow‐related signal enhancement because unlike in 2D imaging where spins enter the imaging slice without prior excitation, the RF excitation in our 3D approach is non‐selective. As a result, the signal is more saturated in the current 3D sequence and the blood‐myocardium contrast appears reduced. A 3D slab‐selective excitation may improve the contrast, by balancing inflow signal enhancement with the need for additional scan planning.

Despite these limitations, 3D acquisitions offer the advantage of simplified scan planning with full coverage and retrospective interrogation of arbitrary planes across multiple organs. This flexibility may be valuable for evaluating complex structures such as the fetal brain, heart, and abdominal organs. Moreover, 3D acquisitions inherently provide contiguous slices with isotropic resolution for more reliable volumetric assessment. While future studies must validate motion‐corrected 3D fetal MRI against 2D approaches, the potential advantages may offset current limitations of 3D fetal MRI, supporting this proof‐of‐concept study and positioning 3D MRI as a promising alternative for future clinical use. Beyond fetal MRI, this approach may extend to neonatal, pediatric, and adult imaging to enhance visualization of cardiac, cerebral, and other anatomies, which require 3D volumetric acquisitions and are prone to bulk motion artifacts.

## 5 Conclusion

A framework (ACROBATIC) that estimates and corrects for displacement of the fetus due to maternal respiration and fetal bulk motion in 3D radial MRI acquisitions has been developed and validated in simulations and in utero. These initial findings hold promise for advancing fetal MRI as a diagnostic tool and continuing the development of fetal‐specific acquisition and reconstruction methods. Further refinements to the 3D acquisition will be essential to evaluate the clinical utility of images reconstructed with ACROBATIC and to address the gap in image quality compared with established 2D frameworks. This is all in keeping with the goal of providing a high‐resolution volumetric assessment of the fetal anatomy for the management of fetal diseases discovered in utero.

## Conflicts of Interest

D.P. is employed by Siemens Healthineers and our research group receives non‐monetary research support from Siemens Healthineers.

## Supporting information


**Figure S1:** Cumulative position error as a function of fNAV ROI size for simulated data. The overall trend is relatively flat with a zoomed‐in insert provided to visualize the small differences across the range of ROI sizes.
**Figure S2:** Cumulative position error as a function of registration ROI size for simulated data. The trend is more pronounced than for the fNAV ROI (Figure S1) with a more well defined optimum.
**Figure S3:** Cumulative position error as a function of real‐time reconstruction bin width. The impact on CPE is relatively flat but with a well‐defined elbow point providing a trade‐off between computational time and motion correction accuracy.
**Figure S4:** Visualization of the ACROBATIC reconstructions of in utero data. The fetal brain and heart are shown for increasing spatial regularization weights using PROST. A value of 0.1 was determined to provide a trade‐off between residual noise and spatial blur.
**Figure S5:** Visualization of two different ROI sizes—(60 mm)^3^: blue, (156 mm)^3^: orange—relative to the fetal anatomy and surrounding maternal anatomy. Three different views are shown (axial, sagittal, coronal). Two exemplary simulated static reconstructions for the fetal brain with simulated mid‐late gestational and late gestational fetus as well as one in utero reconstruction centered on the fetal brain and on the fetal heart.
**Table S1:** Summary of parameter studies.

## Data Availability

The data that support the findings of this study are openly available in Fetal‐XCMR at https://github.com/cwroy/Fetal‐XCMR.
