# Supplementary material for: Automatic Respiratory and Bulk Patient Motion Corrected 3D Fetal MRI
Source: Magn Reson Med. 2025 Nov 4;95(4):1972–85. doi: 10.1002/mrm.70166 (PMC12850569; doi:10.1002/mrm.70166)
Supplement: Supplementary file 1 — Figure S1: Cumulative position error as a function of fNAV ROI size for simulated data. The overall trend is relatively flat with a zoomed‐in insert provided to visualize the small differences across the range of ROI sizes. Figure S2: Cumulative position error as a function of registration ROI size for simulated data. The trend is more pronounced than for the fNAV ROI (Figure S1) with a more well defined optimum. Figure S3: Cumulative position error as a function of real‐time reconstruction bin width. The impact on CPE is relatively flat but with a well‐defined elbow point providing a trade‐off between computational time and motion correction accuracy. Figure S4: Visualization of the ACROBATIC reconstructions of in utero data. The fetal brain and heart are shown for increasing spatial regularization weights using PROST. A value of 0.1 was determined to provide a trade‐off between residual noise and spatial blur. Figure S5: Visualization of two different ROI sizes—(60 mm)3: blue, (156 mm)3: orange—relative to the fetal anatomy and surrounding maternal anatomy. Three different views are shown (axial, sagittal, coronal). Two exemplary simulated static reconstructions for the fetal brain with simulated mid‐late gestational and late gestational fetus as well as one in utero reconstruction centered on the fetal brain and on the fetal heart. Table S1: Summary of parameter studies. [file MRM-95-1972-s001.docx]

# Supporting Information

## Parameter Optimization for ACROBATIC Framework

The following experiments were performed to support the tuning parameter choices outlined in the Methods section for each component of the ACTOBATIC framework. For each tuning parameter, simulation data were used as described in the Methods section and included two gestational ages (mid-late, late), two levels of motion (low, high), and two positions (breech, cephalic). The goal of these experiments was to understand the impact of each tunning parameter and chose a value for subsequent reconstructions and analyses provided in the manuscript.

The cost function for quantifying the impact of each parameter on the simulation data was the cumulative position error (CPE) as described in the methods section, but briefly, it provides a measurement of the difference between the estimated position of the fetus and the simulated ground truth. The regularization weight for PROST-based denoising was evaluated visually using *in utero* data. The following describes each experiment and then summarizes the results and parameter choices for simulated and *in utero* data.

### Correcting Displacement Due to Maternal Respiration Using Focused Navigation (fNAV)

The only tuning parameter for fNAV is the size of the ROI which is a cubical volume extending from the center of the image. Note that the center of the image should correspond to the center of the anatomy of interest which may be the result of how the data were acquired or using the optional manual preparation step described in the Methods section.

On the one hand, ROI sizes that are too large may not be able to decouple displacement of the fetus from other static surrounding tissues. A large ROI also requires additional computation time to perform the NUFFT operation and measure the image quality metric (entropy of the gradient image). On the other hand, ROI sizes that are too small may not contain enough tissue contrast to distinguish the effects of motion blur when measuring the image quality metric.


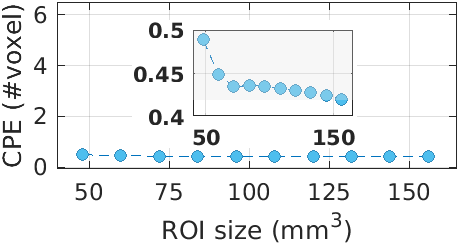


**Figure S1:** **Cumulative position error as a function of fNAV ROI size for simulated data. The overall trend is relatively flat with a zoomed-in insert provided to visualize the small differences across the range of ROI sizes.**

Figure S1 shows the impact of ROI size on the accuracy (CPE) of fNAV. Overall, the ROI size chosen for fNAV did not have a large impact on the CPE. The optimized value is discussed in the following section.

### Correcting Displacement Due to Bulk Motion Using Rigid Registration

The first tuning parameter for bulk motion correction is the ROI size used for registration which is also defined as a cubical volume extending from the center of the image. Similar trade-offs exist for the registration ROI as for the fNAV ROI. A large ROI adds computational time and may not separate moving and static tissue. A small ROI may not have enough tissue contrast to properly co-register images.


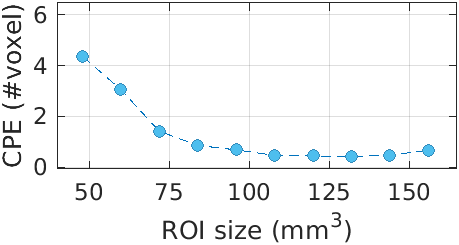


**Figure S2:** **Cumulative position error as a function of registration ROI size for simulated data. The trend is more pronounced than for the fNAV ROI (Figure S1) with a more well defined optimum.**

Figure S2 demonstrate the impact of ROI size on the accuracy of tracking fetal displacement due to bulk motion. Here, the size of the ROI used for registration has a much larger effect than the one used for fNAV. Given that we want consistency between the respiratory and bulk motion components of our framework, we therefore chose the ROI for fNAV, and registration based on Figure S2 and chose the value of (132 mm)^3^ which corresponded to the minimum CPE for simulation data. The second parameter for bulk motion correction is the minimal temporal bin width of real-time images which is defined as the minimal number of radial spokes to include in each bin. A large bin width results in fewer reconstructed frames and faster computation times for both the NUFFT and image registration. However, this may come at the cost of increased intra-bin motion that is not fully captured by the fNAV correction of displacement due to maternal respiration. Conversely, a small bin width may result in too much residual artifact and prevent proper image registration.


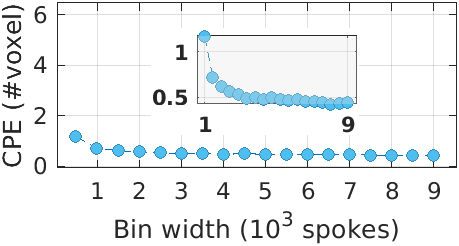


**Figure S3: Cumulative position error as a function of real-time reconstruction bin width. The impact on CPE is relatively flat but with a well-defined elbow point providing a trade-off between computational time and motion correction accuracy.**

Figure S3 shows the impact of different bin widths on the accuracy of tracking displacement due to fetal bulk motion (CPE). Given that the trend was relatively flat, we chose the elbow point value of approximately 1500 spokes to provide a trade-off between accuracy, reconstruction time, and potential residual intra-bin motion. This resulted in approximately 30-40 bins per acquisition with a retrospective real-time temporal resolution of ~8 seconds.

### Reconstructing Motion Corrected 3D Volumes

There are four tuning parameters for PROST denoising as part of the final reconstruction of motion corrected 3D volumes. These include the patch size of 4x4x4 voxels, a search window radius of 20, a number of similar selected patches of 20. These three parameters were kept fixed based on previous work. The spatial regularization weight was determined empirically by visually inspecting reconstruction with different weights and choosing the value that produced modest denoising without noticeable blurring of structures (Figure S4).


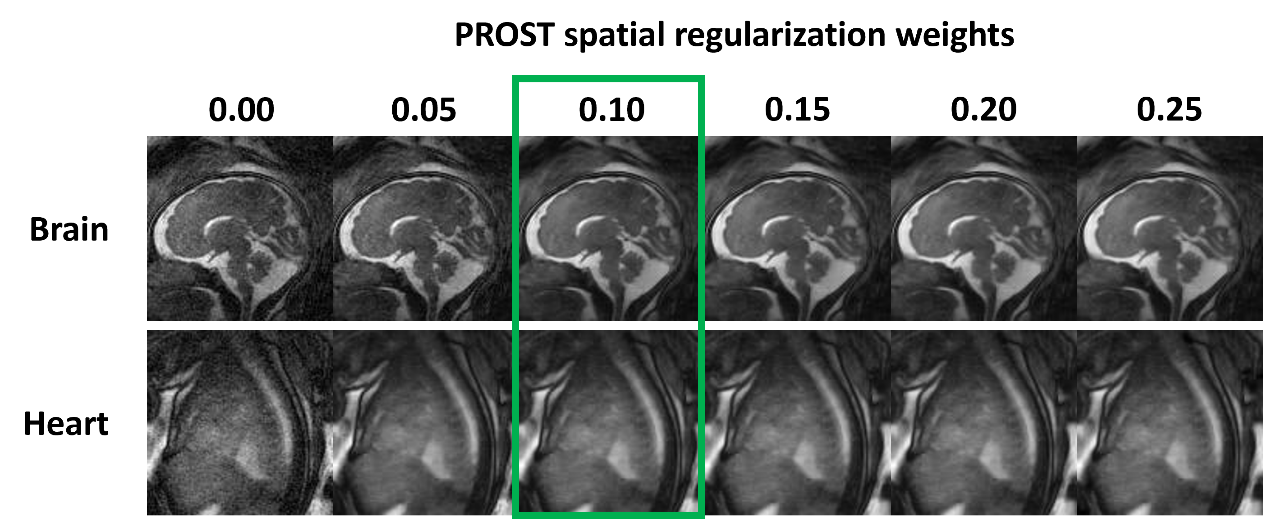


**Figure S4: Visualization of the ACROBATIC reconstructions of in utero data. The fetal brain and heart are shown for increasing spatial regularization weights using PROST. A value of 0.1 was determined to provide a trade-off between residual noise and spatial blur.**

## Summary

The simulation results were invaluable in performing these experiments and arriving at optimum values for the tunning parameters (Table S1). However, we noted differences between the simulation and *in utero* data, in particular, the amount of static amniotic fluid surrounding the simulated fetus versus the proximity of maternal static and moving anatomy to the fetus found in our *in utero* data (Figure S5). We therefore adjusted the ROI for fNAV and registration to be smaller (60 mm)^3^ than what we used for simulated data (132 mm)^3^. This helped with the separation of static and moving anatomy *in utero*.


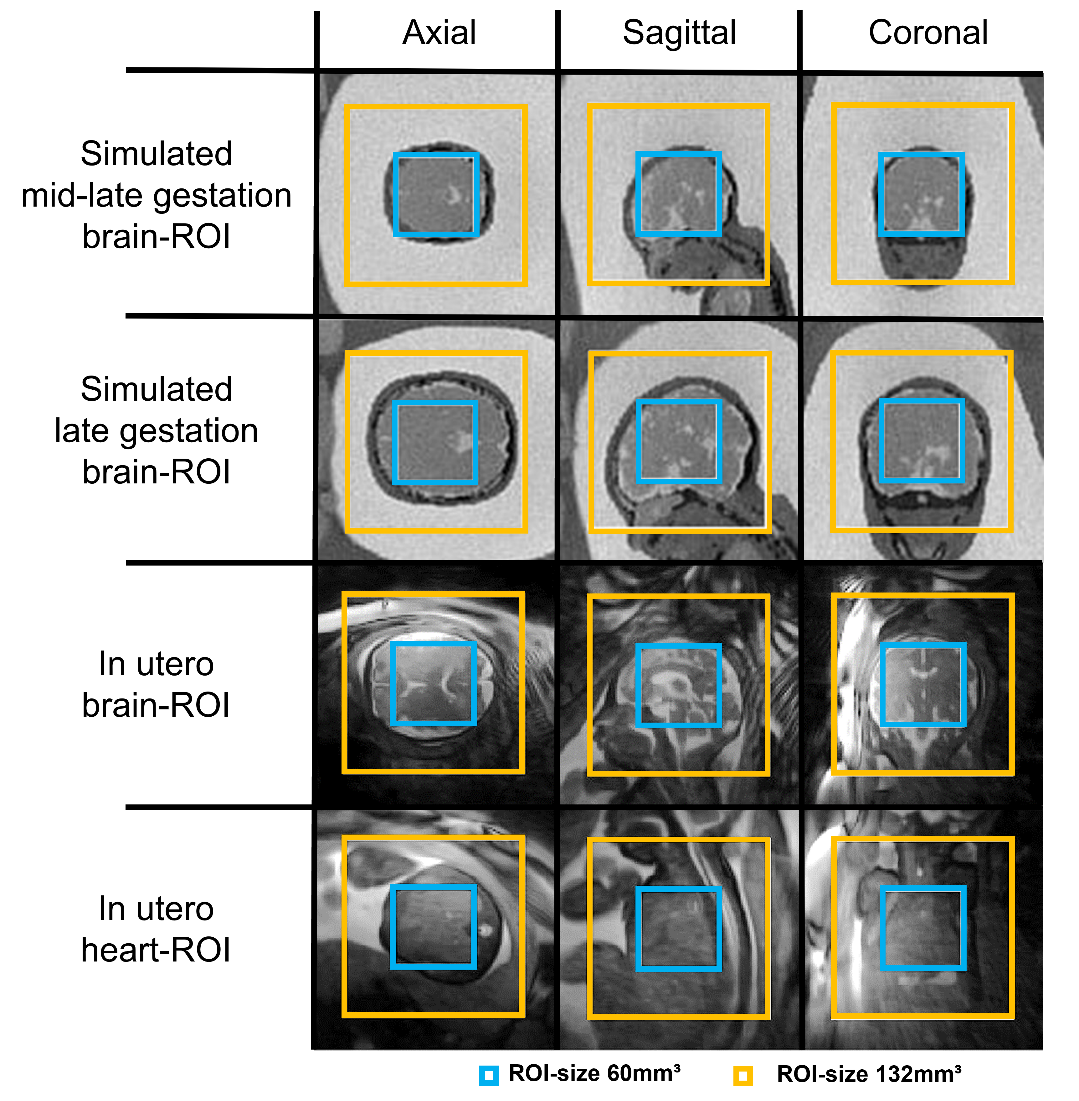


**Figure S5: Visualization of two different ROI sizes - (60mm)³: blue, (156mm)³: orange - relative to the fetal anatomy and surrounding maternal anatomy. Three different views are shown (axial, sagittal, coronal). Two exemplary simulated static reconstructions for the fetal brain with simulated mid-late gestational and late gestational fetus as well as one in utero reconstruction centered on the fetal brain and on the fetal heart.**

**Table S1: Summary of parameter studies.**

| **Parameter** | **In simulations** | ***In utero*** |
| --- | --- | --- |
| ROI size for respiratory motion correction (fNAV) | (132 mm)³ | (60 mm)^3^ |
| ROI size for 3D rigid registration | (132 mm)³ | (60 mm)^3^ |
| Minimal bin width for 3D rigid registration | 1500 spokes ~ 4.3s | 1500 spokes ~ 4.3s |
| PROST spatial regularization weight | 0.1 | 0.1 |
